# Comorbidity of Geo-Helminthes among Malaria Outpatients of the Health Facilities in Ethiopia: Systematic Review and Meta-Analysis

**DOI:** 10.3390/ijerph18030862

**Published:** 2021-01-20

**Authors:** Minyahil Tadesse Boltena, Ziad El-Khatib, Abraham Sahlemichael Kebede, Benedict Oppong Asamoah, Andualem Tadesse Boltena, Melese Yeshambaw, Mulatu Biru

**Affiliations:** 1 Armauer Hansen Research Institute, Ministry of Health, Addis Ababa 1005, Ethiopia; myeshambaw@gmail.com (M.Y.); mulatu.biru@ahri.gov.et (M.B.); 2 Department of Global Public Health, Karolinska Institutet, 171 77 Stockholm, Sweden; ziad.el-khatib@ki.se; 3 World Health Programme, Université du Québec en Abitibi-Témiscamingue (UQAT), Rouyn-Noranda, QC J9X5E4, Canada; 4 School of Health Sciences, University of Brighton, Brighton BN1 9PH, UK; a.s.kebede@brighton.ac.uk; 5 Social Medicine and Global Health, Department of Clinical Sciences, Lund University, 221 00 Lund, Sweden; Benedict_Oppong.Asamoah@med.lu.se (B.O.A.); andy_tadesse@yahoo.com (A.T.B.)

**Keywords:** coinfection/comorbidity, intestinal helminthiases, malaria, outpatient, Ethiopia

## Abstract

Background: Coinfection of malaria and intestinal helminths affects one third of the global population, largely among communities with severe poverty. The spread of these parasitic infections overlays in several epidemiological locations and the host shows different outcomes. This systematic review and meta-analysis determine the pooled prevalence of malaria and intestinal helminthiases coinfections among malaria suspected patients in Ethiopia. Methods: Primary studies published in English language were retrieved using appropriate search terms on Google Scholar, PubMed/MEDLINE, CINHAL, Scopus, and Embase. The Joanna Briggs Institute Meta-Analysis of Statistics Assessment and Review Instrument (JBI-MAStARI) was used for critical appraisal of studies. A pooled statistical meta-analysis was conducted using STATA Version 14.0 software. The heterogeneity and publication bias were assessed using the I2 statistics and Egger’s test, respectively. Duval and Tweedie’s nonparametric trim and fill analysis using the random-effect analysis. The Random effects model was used to estimate the summary prevalence of comorbidity of malaria and soil transmitted helminthiases and the corresponding 95% confidence intervals (CI). The review protocol has registered in PROSPERO number CRD42019144803. Results: We identified ten studies (*n* = 6633 participants) in this study. The overall pooled result showed 13% of the ambulatory patients infected by malaria and intestinal helminths concurrently in Ethiopia. The pooled prevalence of *Plasmodium falciparum* and *Plasmodium vivax*, and mixed infections were 12, 30, and 6%, respectively. The most common intestinal helminth parasites detected were *Hookworm*, *Ascaris lumbricoides*, and *Tirchuris trichiura*. Conclusions: The comorbidity of malaria and intestinal helminths causes lower hemoglobin level leading to maternal anemia, preterm delivery, and still birth in pregnant women and lactating mother. School-aged children and neonates coinfected by plasmodium species and soil transmitted helminths develop cognitive impairment, protein energy malnutrition, low birth weight, small for gestational age, and gross motor delay. The Ministry of Health of Ethiopia and its international partners working on malaria elimination programs should give more emphasis to the effect of the interface of malaria and soil transmitted helminths, which calls for an integrated disease control and prevention.

## 1. Background

Various forms of coinfections occur globally, mostly among societies with high poverty indices. It is projected that more than thirty-three percent of the world’s population living in the tropical and sub-tropical region is mainly infected by malaria and parasitic helminths and Sub-Saharan Africa bears the highest prevalence of Malaria and intestinal helminths comorbidity [1,2,3]. According to the 2019 World Health Organization (WHO) malaria report, an estimated 228 million cases and 405,000 deaths related to malaria in the year 2018 were registered, with the SSA region bearing the highest burden, with 93% of all cases [4,5].

Intestinal helminth infections are the most predominant of lingering human infections and grounded on existing evidence, there are an estimated 1221 million *Ascaris lumbricoides*, 795 million *Trichuristrichiura*, 740 million *Hookworms*, 206.4 million *Schistosoma* spp. [1,6].

Various epidemiological settings have different malaria and intestinal helminthiases coinfections with the host exhibiting varying outcomes [2,7,8,9,10,11,12,13]. Concurrent infection from malaria and helminthes is associated with health complications in the host causing immunosuppression and reduced hemoglobin concentration resulting in anemia [14]. Women infected with soil transmitted helminthiasis are nearly five times more likely to suffer from malaria infection and pregnant mothers coinfected by malaria and geohelminths in malaria endemic regions are at higher risk of feto-maternal morbidity and mortality [12,14,15].

Pregnant women and nursing mothers, neonates and school-aged children are known to be the risk groups affected by the concomitant malaria intestinal helminthic infection [16]. Pregnant women and nursing mothers co-infected by malaria Plasmodium and geo-helminthic parasites are at higher risk for maternal anemia, preterm deliveries, and still births [17,18]. Comorbidity of *Plasmodium* species and soil transmitted helminthic infection causes cognitive impairment, protein energy malnutrition and mild to severe anemia in children [19,20], and low birth weight, small for gestational age and gross motor outcomes in infants [21,22].

Nevertheless, how co-infections affect the epidemiology and pathogenesis of each other is still controversial [23,24,25] ranging from lower severity and lower incidence of malaria to higher severity of malaria in co-infections [26,27,28,29,30,31,32,33,34,35,36,37,38,39]. The fundamental cause for such different outcomes could be attributed to several factors, including variation in defining the case and design of the study, analysis of the data and interpretation, the expression of antigen cross-reactivity between co-infecting organisms and host factors [25].

According to our knowledge, there is a lack of studies on the impact of comorbidity of malaria and soil transmitted helminthes, and its prevalence and severity in Ethiopia. Therefore, this study will determine the pooled magnitude of the comorbidity of malaria and intestinal helminthiasis among the malaria outpatients in Ethiopia.

## 2. Methods

This review was registered in the Prospective International Register of Systematic Reviews (PROSPERO with number CRD42019144812) and is reported according to the MOOSE (Meta-analysis Of Observational Studies in Epidemiology) guidelines [40].

### 2.1. Search Strategy and Selection of Studies

The search strategy was aimed to locate both published and grey literature. An initial limited search of Google Scholar was undertaken to identify articles on the topic. The text words contained in the titles and abstracts of relevant articles, and the index terms used to describe the articles were used to develop a full search strategy for PubMed/Medline, EMBASE, CINHAL, Google Scholar, and Scopus to adapted for each included information source. A combination of keywords and systematic search term including all identified keywords and index terms, were: (((“Coinfection”[MeSH Terms] OR “Comorbidity”[MeSH Terms] OR “Intestinal helminthiasis”[Supplementary Concept]) AND “Malaria”[MeSH Terms]) OR (“Plasmodium vivax”[MeSH Terms] OR “Plasmodium falciparum”[MeSH Terms] OR “Plasmodium malariae”[MeSH Terms]) OR “Pregnant Women”[MeSH Terms]) AND (“humans”[MeSH Terms] AND “english”[Language]) AND ((((“coinfection*”[Text Word] OR “comorbidity*”[Text Word] OR “intestinal helminthiasis*”[Text Word] OR “soil transmitted helminthiases*”[Text Word] OR “geohelminth*”[Text Word]) AND “malaria*”[Text Word]) OR “plasmodium vivax*”[Text Word] OR “plasmodium falciparum*”[Text Word] OR “plasmodium malariae*”[Text Word] OR “pregnant women*”[Text Word] OR “pregnant mother*”[Text Word]) AND (“humans”[MeSH Terms] AND “english”[Language])) used to identify studies. 

The reference list of all studies selected for critical appraisal was screened for additional studies. Institution-based cross-sectional studies published in the English language were included. Literature was eligible for inclusion if they reported the magnitude of comorbidity of intestinal helminthiases among malaria outpatients in Ethiopia. Systematic reviews and studies found to have methodological flaws after a quality assessment were excluded.

**Figure 1:**
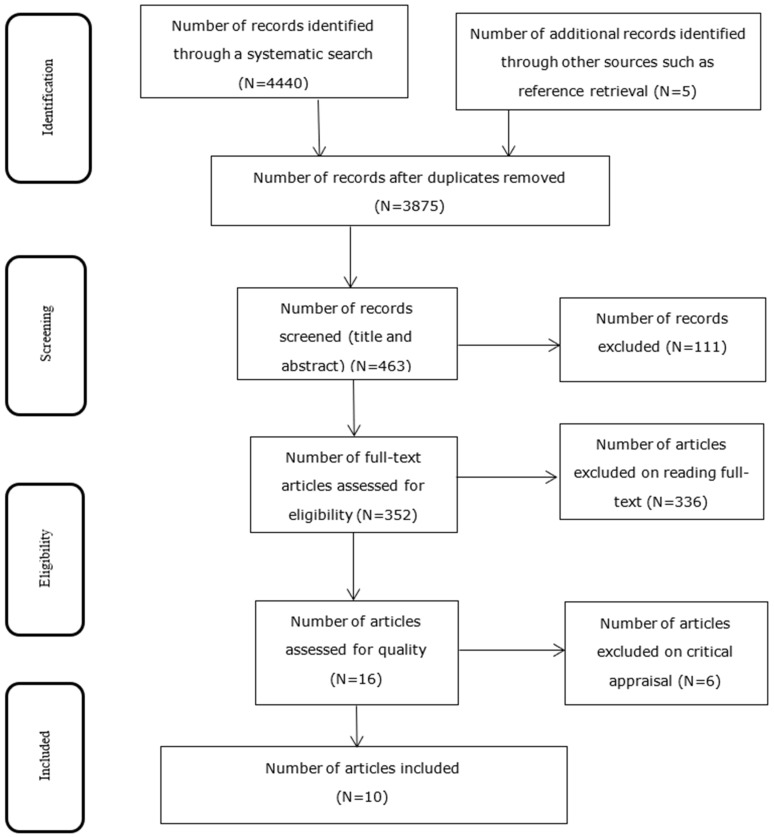
PRISMA flow chart diagram describing studies selected for systematic review and meta-analysis of comorbidity of geo-helminthes and malaria among outpatients in Ethiopia, 2020.

Following the search, all identified citations were organized and uploaded into EndNote version 15.0 and duplicates were removed. Titles and abstracts were screened by two independent reviewers (MTB and MBS) and cross checked by a third reviewer (MY) for the assessment against the inclusion criteria for the review. Potentially relevant studies were retrieved in full including their citation details. The full text of selected citations was assessed in detail against the inclusion criteria by two reviewers (MTB and MBS) and double-checked by another independent reviewer (MY). Reasons for exclusion of full-text studies that do not meet the inclusion criteria were recorded and reported in the systematic review. Any disagreements that arise between the reviewers at each stage of the study selection process were resolved through discussion, or with a third reviewer. The results of the search were reported in full in the final systematic review and presented in a Preferred Reporting Items for Systematic Reviews and Meta-analyses (PRISMA) flow diagram Figure 1 [41].

Definition of malaria and helminthic co-infection: Clinically confirmed co-infection developed by either of the *Plasmodium* species and one or more of the intestinal/soil transmitted helminthes.

### 2.2. Data Extraction and Management

The authors jointly prepared and determined the data extraction tool for this study. The data were extracted from primary studies included in the review using the data extraction tool prepared by two independent reviewers. The tool included variables such as the name of the author, publication year, study design, data collection period, sample size, study area/region, age with the highest malaria infection, prevalence of *Plasmodium falciparum* infection, *Plasmodium vivax* infection, and mixed infection and magnitude of coinfection of malaria and soil transmitted helminthes. Additionally, the tool contained information on the tools used for the diagnosis of malaria and intestinal helminthiases. Two authors (MTB and MBS) were involved in the data extraction; any disagreements that arose between the reviewers were resolved through discussion, or with a third reviewer. Authors of papers were contacted to request missing or additional data if needed.

The required information from each primary study was extracted by using a format prepared in a Microsoft Excel spreadsheet. Eligible studies were critically appraised by two independent reviewers (MTB and MBS) at the study level for methodological quality in the review using standardized critical appraisal instruments from the Joanna Briggs Institute (JBI) for incidence and prevalence [42]. Authors of papers were contacted to request missing or additional data for clarification where required. Any disagreements that arose were resolved through discussion, or with a third reviewer (MY). Following the critical appraisal, studies that did not meet certain a quality threshold were excluded. This decision was based on inadequate sample size, inappropriate sampling frame, and data analysis conducted with sufficient coverage of the identified sample (Table 1). Articles were reviewed using titles, abstracts, and full text review. Studies that did not meet inclusion criteria were excluded.

### 2.3. Data Synthesis and Analysis

**Figure 2:**
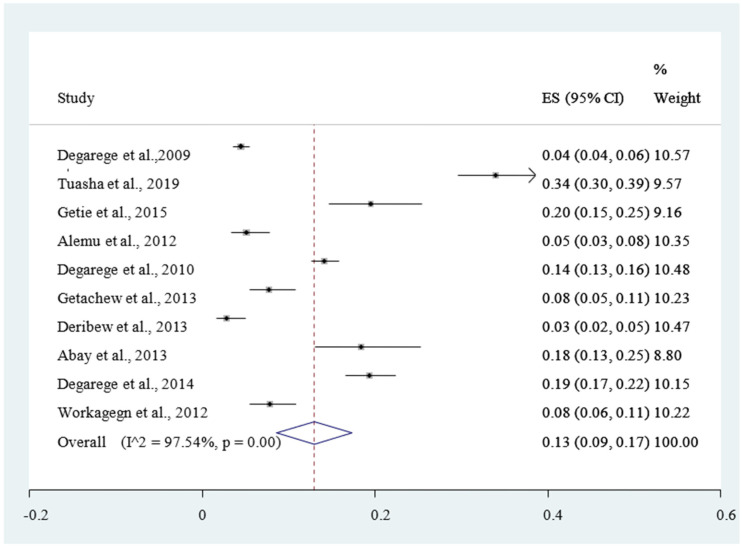
Forest plot of the pooled estimate of the comorbidity of geo-helminths and malaria among outpatients in Ethiopia, 2020.

**Figure 3:**
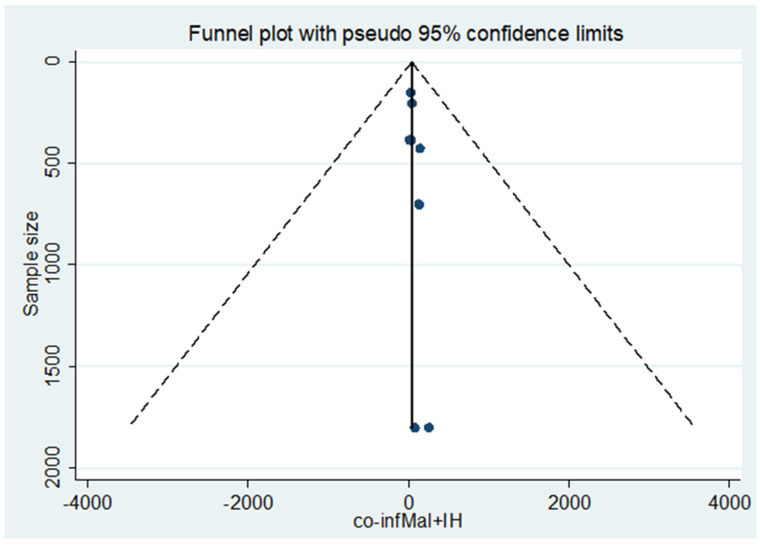
Funnel plot with 95% confidence limit of the comorbidity of geo-helminths and malaria among the outpatients in Ethiopia, 2020.

Included studies were pooled in a statistical meta-analysis using STATA version 14. Effect sizes were expressed as a proportion with 95% confidence intervals around the summary estimate. Heterogeneity was assessed statistically using the standard chi-square *I*^2^ test. A random-effects model using the double arcsine transformation approach was used. Sensitivity analyses were conducted to test decisions made regarding the included studies and to get the effect of a single study on the total estimation Figure 2. Forest plots with 95% CI were compute to estimate the pooled magnitude of comorbidity of malaria and intestinal helminthes among outpatients in the health facilities of Ethiopia Figure 2. Visual examination of funnel plots asymmetry Figure 3 and Egger’s regression tests were used to check for publication bias [43].

## 3. Results

### 3.1. Search

A total of 4445 research articles were identified by electronic search in MEDLINE/PubMed, Google Scholar, CINAHL, EMBASE, and Scopus databases. Of these, 570 were excluded due to duplication, 3412 through review of titles and abstracts. Additionally, 352 studies found to be eligible for full-text screening, out of which 336 were excluded for not reporting the outcome variable (prevalence of comorbidity of malaria and geo-helminthes among outpatients of the health facilities in Ethiopia). A total of 16 studies were eligible for quality assessment, and finally 10 studies were found to be eligible and included in the meta-analysis Figure 1.

### 3.2. Included Study Characteristics

The total sample size of the included studies in this review were 6633 patients, ranging among studies included from 152 in Oromia region [44] to 1802 in Southern Nations, Nationalities, and Peoples’ Region (SNNP) [45]. Six of the included studies were from SNNP region [45,46,47,48,49,50], two studies from Amhara region [51,52], one from Afar region [53] and one from Oromia region [44]. The study design of all included studies in this review was cross-sectional (Table 2).

### 3.3. Pooled Prevalence of Malaria and Intestinal Helminthiases

**Figure 4:**
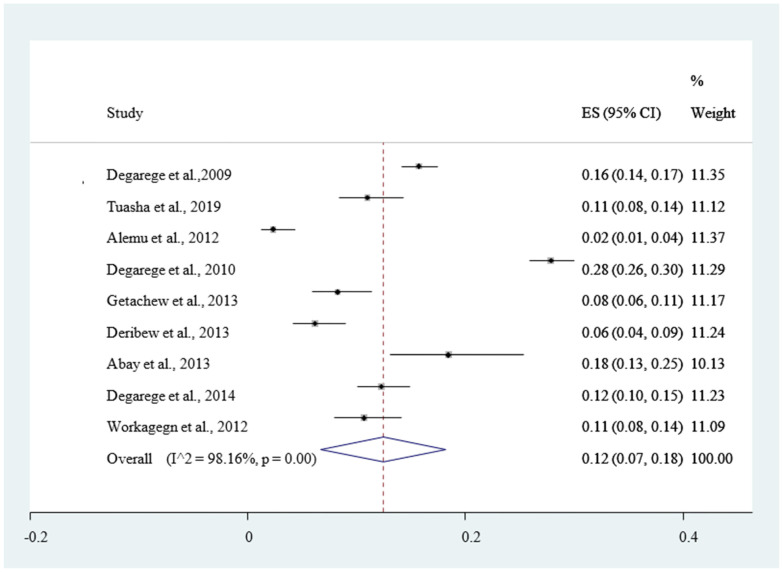
Forest plot of 9 studies on magnitude of *Plasmodium falciparum* infection among outpatients in Ethiopia, 2020.

**Figure 5:**
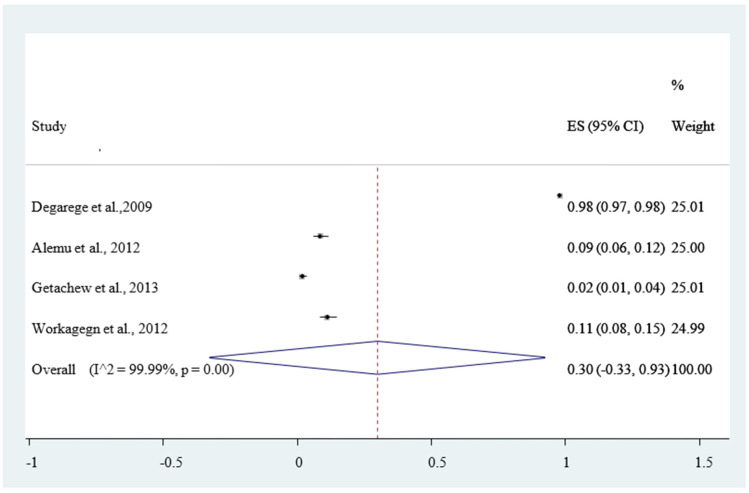
Forest plot of 4 studies on magnitude of *Plasmodium vivax* infection among out patients in Ethiopia, 2020.

**Figure 6:**
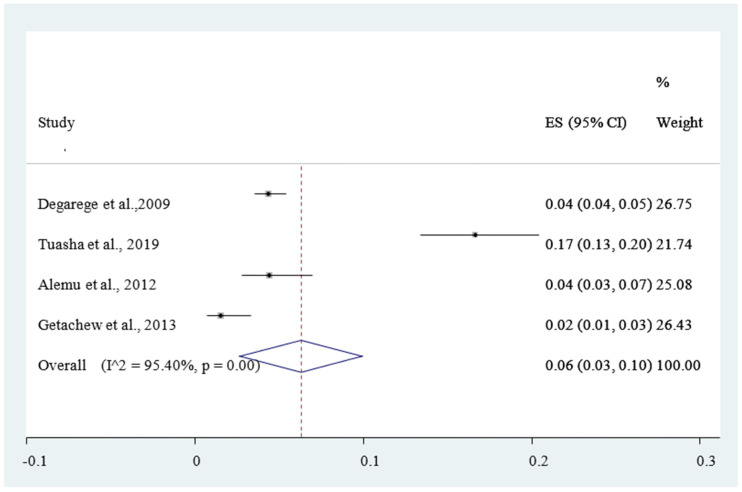
Forest plot of 4 studies on magnitude of *Plasmodium falciparum* and *Plasmodium vivax* mixed infection among outpatients in Ethiopia, 2020.

**Figure 7:**
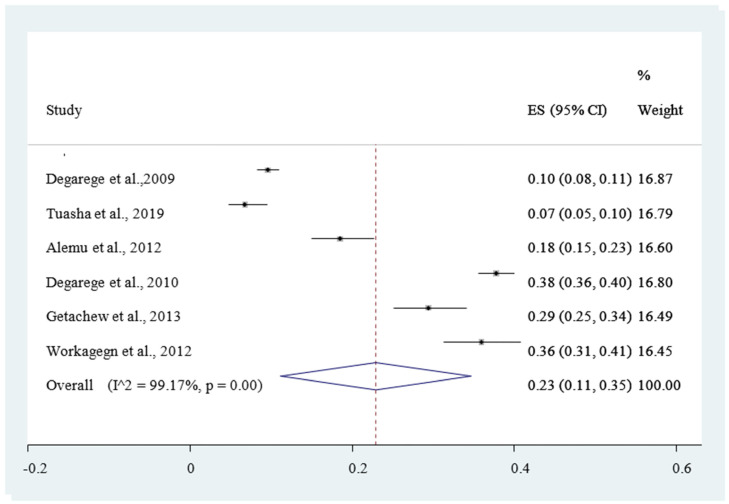
Forest plot of 6 studies on magnitude of *Hookworm* infection among outpatients in Ethiopia, 2020.

**Figure 8:**
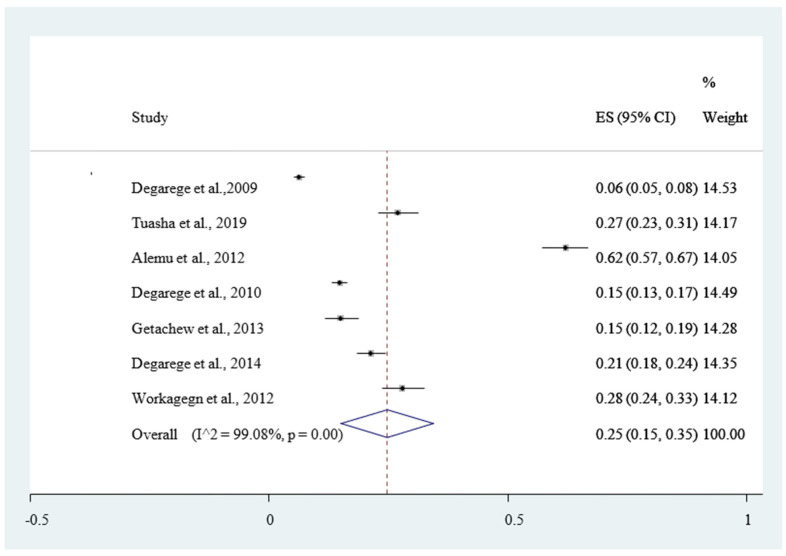
Forest plot of 7 studies on magnitude of *Ascaris lumbricoides* infection among outpatients in Ethiopia, 2020.

**Figure 9:**
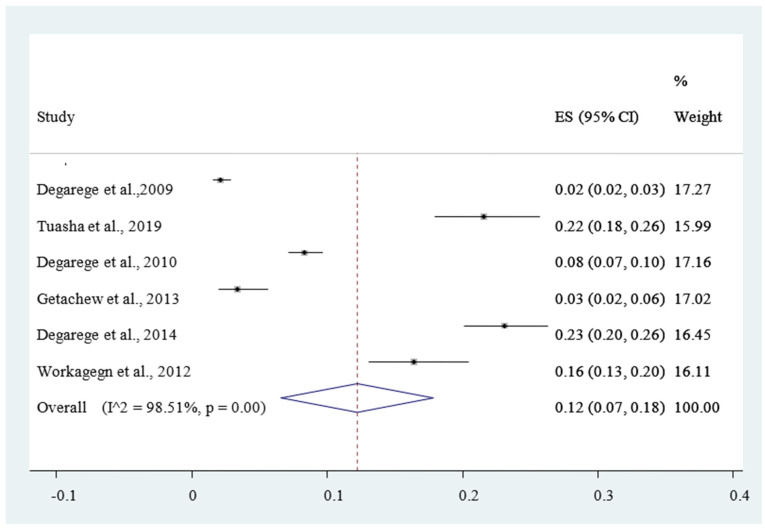
Forest plot of 6 studies on magnitude of *Trichuris trichiura* infection among outpatients in Ethiopia, 2020.

The magnitude of concomitant malaria and intestinal helminthic infection varied with different geographical locations across Ethiopia, with the lowest co-infection (2.84%) observed in Eastern Ethiopia [53] whereas the highest (55.7%) was reported in Southern Ethiopia [47]. The pooled prevalence of *P. falciparum*, *P. vivax*, and mixed infection (*P. falciparum* and *P. vivax* co-infection) were 12% Figure 4, 30% Figure 5, and 6% Figure 6, respectively. The *I*^2^ test statistics result showed significant heterogeneity (*I*^2^ = 98.16%, *p* < 0.001) for *P. falciparum* Figure 4, (*I*^2^ = 99.99%, *p* < 0.001) for *P. vivax* Figure 5, and (*I*^2^ = 95.40%, *p* < 0.001) for mixed infection from both *P. falciparum* and *P. vivax*, with Eggers test (*p* < 0.001) for all indicates that the publication bias was not found. The Duval and Tweedie’s nonparametric trim and fill analysis using the random-effect analysis was conducted to account for publication bias and heterogeneity revealed that the *I*^2^ test result showed high heterogeneity (*I*^2^ = 99.99%, *p* < 0.001) but Egger’s test showed no statistically significant publication bias. The pooled magnitude of intestinal helminthic infection was 23% *Hookworm* Figure 7, 25% *Ascaris lumbricoides* Figure 8, and 12% *Trichuris trichiura* Figure 9.

Therefore, the Duval and Tweedie nonparametric trim and fill analysis using the random-effect analysis was conducted to account for publication bias and heterogeneity. Accordingly, the pooled result of the final eligible studies for meta-analysis showed 13% (95% CI: 12%, 26%) of malaria and intestinal helminthic comorbidity among the outpatients in Ethiopia 

Figure 2, The distribution of intestinal helminthic infection was highly prevalent in SNNP region. *Hookworm* (37.8%), *Tirchuristrichiura* (64.5%), and *Schistosoma mansoni* (28.4%) and an infection from *Ascaris lumbricoides* (62.1%) were widely distributed in Amhara region (Table 2). Children aged less than five years were highly infected by malaria parasite (39%) (Table 2). The microscopic technique and Kato–Katz thick smear testing were the two commonly used methods used to detect the malaria parasites and intestinal helminthic infection, respectively (Table 2).

## 4. Discussion

Data from 10 studies conducted in four different regions of Ethiopia were analyzed to determine the pooled prevalence of comorbidity of malaria and intestinal helminthiases among the outpatients. These studies reported results on the coinfection of malaria and intestinal helminths, distribution of malaria parasitemia, and intestinal helminthic infection across the regions for a total of 6633 outpatients. 

The finding of this review showed that 13% (95% CI: 9–17%) of the ambulatory patients were infected by the malaria and intestinal helminths. This finding was higher than a study conducted in Tanzania (5%) and Nigeria (12%) [54,55]. However, it is lower than a study conducted in peri-urban community in Nigeria (63%) [56]. This difference could be attributed to the effective control and prevention mechanism better applied in Nigeria and Tanzania as compared to Ethiopia [54,55,56].

Outpatients including pregnant women and lactating mothers co-infected by *Plasmodium* species and geo-helminthes are at higher risk of anemia, preterm birth, and death or loss of a baby before or during delivery [57,58,59]. School-aged children co-infected by both malaria and helminthic parasites may develop impaired memory, deficiency in micronutrients essential for growth and development and mild to severe anemia [60,61]. Co-infection may also result in babies born weighing less than 2.5 kg or an infant smaller or less developed than normal for the baby’s sex and gestational age and gross motor outcomes in infants [62,63].

Reports of nine articles were used to estimate the pooled prevalence of *Plasmodium falciparum* 12% (95% CI: 7–18%). This finding was higher than a report from systematic review and meta- analysis where the pooled magnitude of *P. falciparum* infection was 1.4% globally [64], and lower than study conducted in Ethiopia were the pooled *Plasmodium falciparum* infection was 14.7% (95% CI: 21.3, 30.4) [65].

In our report, four studies analyzed the pooled estimate of *Plasmodium vivax* infection. According to this study, the pooled magnitude of the *P. vivax* infection among the outpatients were found to be 30% (95% CI: 33–93%). This finding was higher than a study conducted in Ethiopia 8.7% [65] and lower than a systematic review and meta-analysis 38% [66]. This difference could be attributable to the parasite density and demographic variations [67].

Findings from four eligible primary studies of this review were used to analyze the pooled prevalence of the mixed *Plasmodium* infection, which was found to be 6% (95% CI: 3–10%). This finding is lower than a report from systematic review and meta-analysis of *Plasmodium* spp. Mixed infection 9% (95% CI: 7.0–12.0%) [68] and a similar study reported from Ethiopia 25.8% (95% CI: 21.3–30.4%) [65].

Patients infected with *Plasmodium* species may develop severe complications such as severe anemia, pulmonary complications, renal failure, impaired consciousness, and jaundice. In total, six studies out of the ten eligible articles have reported to estimate the pooled prevalence of hookworm infection in the outpatients. A total of 23% (95% CI 11–35%) of the study participants were infected by hookworm.

This result is in agreement with a systematic review and meta-analysis in Nigeria [69]. However, this finding is higher than the finding of a systematic review and meta-analysis carried out in Asia [70] and Ethiopia [71]. Despite the differences in the pooled magnitude of hookworm infection, Ethiopia has to better practice hookworm infection controlling strategies in place.

From the eligible studies of this review, seven of them were used to estimate the pooled magnitude of intestinal helminthic infection from *Ascaris lumbricoides* 25% (95% CI 15–35%). This result is in agreement with a report of systematic review from Asia [70] is higher than a global prevalence of *Ascaris lumbricoides* 17% [95% CI 13–21%] [72] and a study conducted in Bangladesh 23% [73]. This variation could be attributable to the poor performance of water and sanitation program and inadequate health education service at the community level in Ethiopia.

According to this study, the pooled prevalence of *Trichuristrichiura* were found to be 12% (95% CI 7–18%). This finding is higher than a national estimate (5.9%) [74] and lower than the finding a systematic review conducted in South Asia and South East Asia [75].

## 5. Conclusions

The comorbidity of malaria and intestinal helminthes causes lower hemoglobin leading to maternal anemia, preterm delivery and still birth in pregnant women. School-aged children and neonates co-infected by *Plasmodium* species and soil transmitted helminthes may develop cognitive impairment, protein energy malnutrition, low birth weight, small for gestational age, and gross motor delay. The Ministry of Health of Ethiopia and its international partners working on malaria elimination programs should give more emphasis to the effect of the interface of malaria and soil transmitted helminths, which calls for an integrated disease control and prevention.

## Figures and Tables

**Table 1:** The quality assessment of the studies included for the pooled estimate of comorbidity of geo-helminthes and malaria among the outpatients in Ethiopia.

	Quality Assessment Criteria Probing Questions (Q)									Study Level Bias Score		Judgment
Included Studies	Q-1	Q-2	Q-3	Q-4	Q-5	Q-6	Q-7	Q-8	Q-9	Total No Yes (Y)	Percentage of Yes (Y)	
Degarege et al. 2009	Y	Y	Y	Y	Y	Y	Y	Y	Y	9	100.0%	Low
Tuasha et al. 2019	Y	Y	Y	Y	U	Y	Y	Y	Y	8	88.9%	Low
Getie et al. 2015	Y	Y	Y	Y	Y	Y	U	Y	Y	8	88.9%	Low
Alemu et al. 2012	Y	Y	Y	Y	U	U	Y	Y	Y	7	77.8%	Moderate
Degarege et al. 2010	Y	Y	Y	U	Y	Y	Y	Y	Y	9	100.0%	Low
Getachew et al. 2013	Y	Y	Y	Y	Y	U	Y	Y	Y	8	88.9%	Low
Deribew et al. 2013	Y	Y	Y	Y	U	Y	U	Y	Y	7	77.8%	Moderate
Abay et al. 2013	Y	Y	Y	Y	Y	Y	Y	Y	Y	9	100.0%	Low
Degarege et al. 2014	Y	Y	Y	Y	Y	U	Y	Y	Y	8	88.9%	Low
Workagegn et al. 2012	Y	Y	Y	Y	Y	Y	U	Y	U	7	77.8%	Moderate
Subtotal												
Y = Yes		89%										
U = Unclear		11%										
N = No		0%										
Overall risk of bias assessment score 89%												

Remark: The risk of bias for each eligible study is calculated from the domain of nine criteria.

**Table 2:** Summary characteristics of 10 studies included in the systematic review and meta-analysis of comorbidity of geo-helminthes and malaria among outpatients in Ethiopia.

S. No	Author, Year of Publication	Year Study Conducted	Region	Study Design	Sample Size	*P.f*	*P.v*	Mixed	Co-Inf Mal+ IH	*Hw*	*Al*	*Tt*
1	Degarege et al., 2009	November and December 2007	SNNPR	Cross-sectional	1802	283	1764	79	81	173	113.5	37.8
2	Tuasha et al., 2019	December 2009 to July 2010	SNNPR	Cross-sectional	427	47		71	145	29	115	92
3	Getie et al., 2015	February to May 2013	Amhara	Cross-sectional	205	147	53	5	40			
4	Alemu et al., 2012	February to March 2011	Amhara	Cross-sectional	384	9	33	17	19.6	71	238.5	
5	Degarege et al., 2010	November and December 2007	SNNPR	Cross-sectional	1802	502			255	681	267	149.6
6	Getachew et al., 2013	August to September, 2011	SNNPR	Cross-sectional	388	45	275.8	51.6	30	114	58	13
7	Deribew et al., 2013	November to December 2008	Afar	Cross-sectional	387	24			11			
8	Abay et al., 2013	November to December 2009	Oromia	Cross-sectional	152				28		149.5	162
9	Degarege et al., 2014	December 2010 to February 2011	SNNPR	Cross-sectional	702	86			136		149.5	162
10	Workagegn et al., 2012	November 2010 to January 2011	SNNPR	Cross-sectional	384	187	197		30	138	107	63

*Al*: *Ascaris lumbricoides*; *Hw*: *Hookworm*; O-Inf Mal+ IH: Coinfection of malaria and intestinal helminthes; *P.f*: *Plasmodium falciparum*; *P.v*: *Plasmodium vivax*; SNNPR: Southern Nations, Nationalities and Peoples Region; *Tt*: *Trichuris trichiura*.

## Data Availability

The datasets during and/or analyzed during the current study are available from the corresponding author on reasonable request.

## Abbreviations

AHRI	Armauer Hansen Research Institute
EPHI	Ethiopian Public Health Institute
JBI	The Joanna Briggs Institute
JBI-MAStARI	The Joanna Briggs Institute Meta-Analysis of Statistical Assessment and Review Instrument
LMICs	Low- and Middle-Income Countries
MOH	Ministry of Health
PRISMA	Preferred Reporting Items for Systematic Reviews and Meta-analyses
PROSPERO	International Prospective Registry of Systematic Reviews
SDG	Sustainable Development Goal
SNNPR	Southern Nations Nationalities and Peoples Region
STH	Soil Transmitted Helminthiases
SSA	Sub-Saharan Africa
WHO	The World Health Organization
